# Single-cell analyses of metastatic bone marrow in human neuroblastoma reveals microenvironmental remodeling and metastatic signature

**DOI:** 10.1172/jci.insight.173337

**Published:** 2024-02-15

**Authors:** Shenglin Mei, Adele M. Alchahin, Bethel Tesfai Embaie, Ioana Maria Gavriliuc, Bronte Manouk Verhoeven, Ting Zhao, Xiangyun Li, Nathan Elias Jeffries, Adena Pepich, Hirak Sarkar, Thale Kristin Olsen, Malin Wickström, Jakob Stenman, Oscar Reina-Bedoya, Peter V. Kharchenko, Philip J. Saylor, John Inge Johnsen, David B. Sykes, Per Kogner, Ninib Baryawno

**Affiliations:** 1 Center for Regenerative Medicine, Massachusetts General Hospital, Boston, Massachusetts, USA.; 2 Department of Biomedical Informatics, Harvard Medical School, Boston, Massachusetts, USA.; 3 Childhood Cancer Research Unit, Department of Women’s and Children’s Health, Karolinska Institutet, Stockholm, Sweden.; 4 Harvard Stem Cell Institute, Cambridge, Massachusetts, USA.; 5 Department of Stem Cell and Regenerative Biology, Harvard University, Cambridge, Massachusetts, USA.; 6 Massachusetts General Hospital Cancer Center, Harvard Medical School, Boston, Massachusetts, USA.

**Keywords:** Bone biology, Oncology, Bone marrow, Cancer

## Abstract

Neuroblastoma is an aggressive pediatric cancer with a high rate of metastasis to the BM. Despite intensive treatments including high-dose chemotherapy, the overall survival rate for children with metastatic neuroblastoma remains dismal. Understanding the cellular and molecular mechanisms of the metastatic tumor microenvironment is crucial for developing new therapies and improving clinical outcomes. Here, we used single-cell RNA-Seq to characterize immune and tumor cell alterations in neuroblastoma BM metastases by comparative analysis with patients without metastases. Our results reveal remodeling of the immune cell populations and reprogramming of gene expression profiles in the metastatic niche. In particular, within the BM metastatic niche, we observed the enrichment of immune cells, including tumor-associated neutrophils, macrophages, and exhausted T cells, as well as an increased number of Tregs and a decreased number of B cells. Furthermore, we highlighted cell communication between tumor cells and immune cell populations, and we identified prognostic markers in malignant cells that are associated with worse clinical outcomes in 3 independent neuroblastoma cohorts. Our results provide insight into the cellular, compositional, and transcriptional shifts underlying neuroblastoma BM metastases that contribute to the development of new therapeutic strategies.

## Abstract 2

Single-cell RNA-sequencing identifies changes in immune cell populations and gene expression profiles in the neuroblastoma bone marrow metastatic niche, providing insight for potential therapeutic interventions.

## Graphical abstract

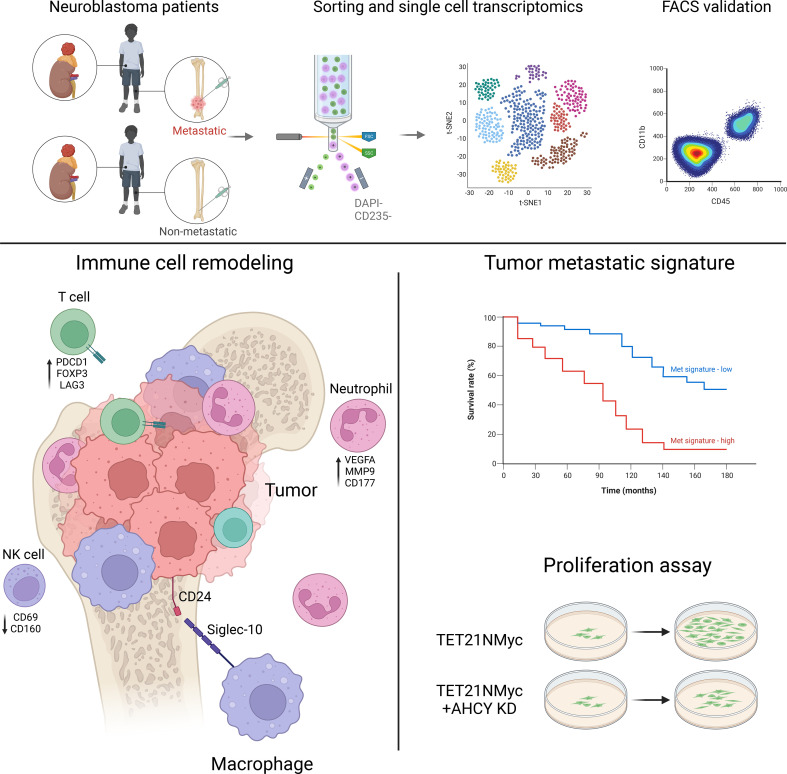

## Introduction

Neuroblastoma (NB) is the most common pediatric extracranial solid tumor that develops from neural crest-derived cells and arises in the developing peripheral sympathetic nervous system (1). At the time of diagnosis, approximately 50% of the patients have metastasis, and more than 90% of patients with metastatic disease have disseminated tumor cells within the bone and BM (2). The 5-year overall survival for patients with localized and low-risk NB is 90% (3), in contrast to 50% for patients with high-risk NB, and survival outcomes drop to less than 10% for patients with relapsed metastatic disease (4, 5). Despite the prevalence of bone metastases in patients with NB, little is known about the mechanism of BM invasion in NB.

Previous single-cell RNA-Seq (scRNA-Seq) studies have unraveled the cell identities, phenotypes, and gene regulatory networks of tumor cells in primary NB (6–9). The neural crest–sympathoadrenal development, the lineage from which NB is thought to originate, has also been elucidated by lineage tracing studies in mice and by scRNA-Seq of murine and human fetal adrenal gland tissue (7, 9). Deciphering NB tumor cell heterogeneity is important to fully understand their metastatic potential (10).

Another important aspect in understanding disease progression and metastasis lies in elucidating the immune cells infiltrating the tumors. Clinical reports have demonstrated therapy resistance in NB to T cell infiltration (11), while it remains unclear whether certain T cell subtypes have prognostic value (12). Additionally, clonal expansion was only revealed in a small number of untreated patients with NB by performing T receptor sequencing (13, 14), indicating that only a limited number of patients may present with tumor antigen response. Preclinical and clinical observations associated myeloid-derived suppressor cells (MDSCs) with poor prognosis (15, 16). The observations of the role that immune cells have in NB contributed to the foundation of immunotherapy resulting in the addition of anti-GD2 immunotherapy as a combination with the standard treatment of patients with high-risk NB (17). While immune cell populations have been investigated in NB, most studies have focused on the primary tumor (2, 15) or were restricted to specific immune cell subtypes (18). A systemic characterization of metastatic BM in patients with NB, as well as comparison of the metastatic tumor versus the primary tumor at single-cell resolution, is still lacking.

Tumor metastasis is a complex and dynamic process that involves genetic and epigenetic alterations, cell-to-cell interactions, and microenvironment changes. Both tumor cells and the host microenvironment play important roles in the metastatic cascade. Multiple immune cell populations are enriched in the BM niche (19) and have been shown to be involved in cancer progression (20). The niche may favor quiescence and promote immune escape, allowing for tumor progression and metastasis (21). In this study, we used single-cell transcriptomics to characterize the NB BM metastatic microenvironment, performed a comparative analysis between nonmetastatic and metastatic BM from patients with NB, and highlighted potential key cellular populations and transcriptional changes involved in NB progression and metastasis. Our data provide an improved understanding of the NB BM metastatic microenvironment that can be exploited for future therapies.

## Results

### Single-cell transcriptomic profiling of BM from patients with NB, with and without metastases.

**Figure 1:**
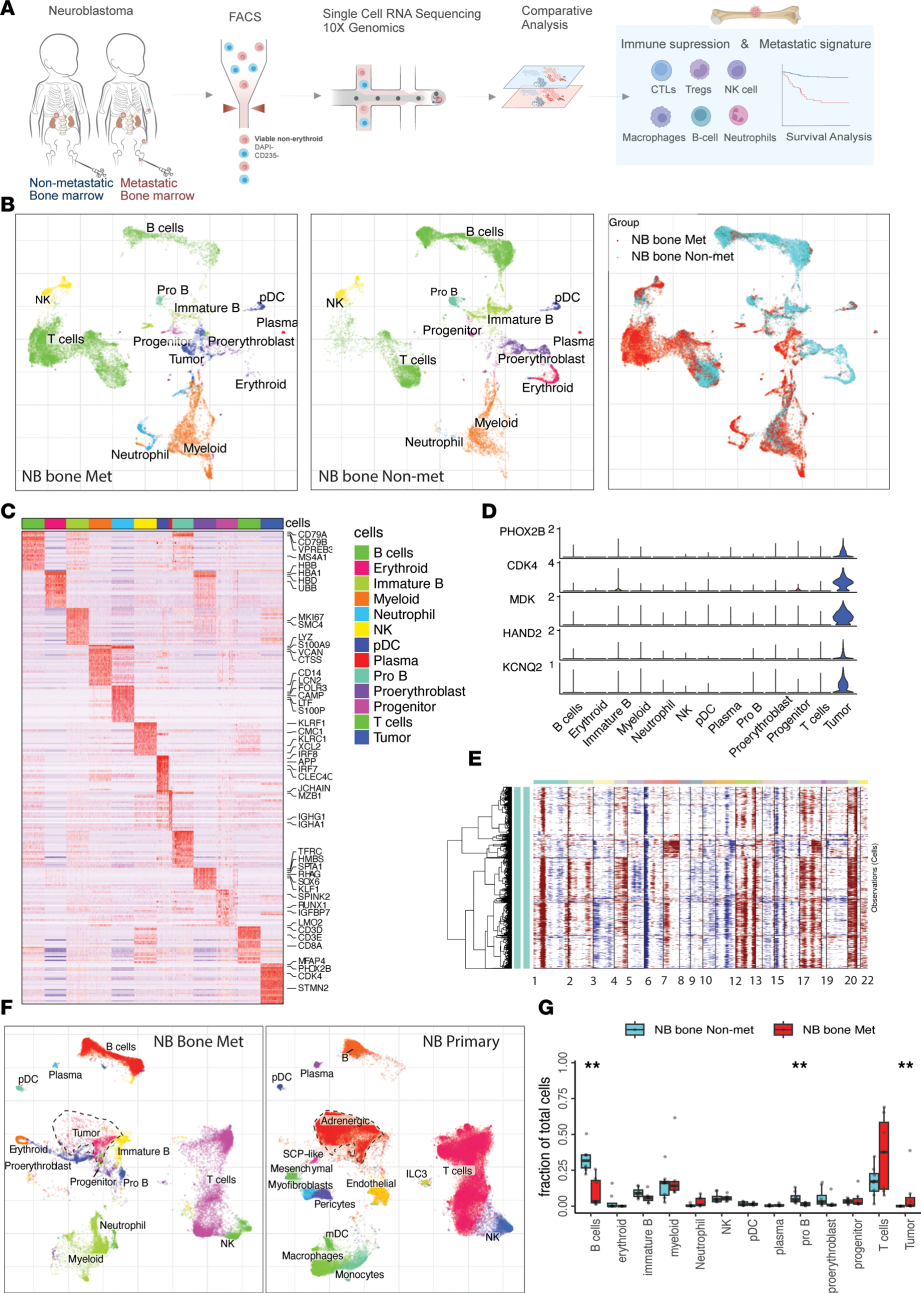
Overview of tumor microenvironment of neuroblastoma nonmetastatic and bone metastatic tumors. (**A**) An illustration depicting the experimental design. (**B**) Integrative analysis of scRNA-Seq samples from 15 NB BM samples visualized using a common UMAP embedding for NB bone metastatic (left), NB bone nonmetastatic samples (middle), and sample fraction. (**C**) Heatmap showing expression of markers for major cell populations. (**D**) Violin plot showing representative marker gene expression for tumor cells. (**E**) Inferred CNV profile of tumor cells from NB bone metastatic tumor. (**F**) UMAP embedding showing joint integration of cells from NB bone metastatic tumor (left; *n* = 15) and NB primary tumor (middle; *n* = 17). (**G**) Box plot comparing proportion of major cell populations between metastatic (*n* = 8) and non- metastatic (*n* = 7) samples. Significance was assessed using 2-sided Wilcoxon ranked-sum test (*P < 0.05, **P < 0.01). For box plots, the center line represents the median, box limits represent upper and lower quartiles, and whiskers depicts 1.5 × the interquartile range (IQR).

To elucidate the changes in BM microenvironment cellular composition that accompany NB metastases (compared with the BM of patients with nonmetastatic NB), fresh patient tissue samples were systematically collected for cell isolation, FACS, and transcriptomic analysis. We used the 10X Genomics platform to profile the single-cell transcriptome, including 7 nonmetastatic and 8 metastatic BM samples (Figure 1A). Metastatic disease, or lack of tumor cells in the BM, was confirmed by pathologic evaluation of stained sections. Detailed clinicopathological information is provided in Supplemental Table 1 (supplemental material available online with this article; https://doi.org/10.1172/jci.insight.173337DS1). After data quality control and doublets removal, 37,406 cells were retained for subsequent analysis, in which 19,565 cells originated from nonmetastatic BM samples and 17,841 cells originated from BM metastatic samples. scRNA-Seq data were integrated using Conos (22). We projected the cells to a unified Uniform Manifold Approximation and Projection (UMAP) embedding space and then performed graph-based clustering. Major cell populations were annotated with their respective markers (Supplemental Table 3).

The cells were classified into 13 major cell types (Figure 1B), including T cells (*CD3D*, *CD3E*), NK cells (*KLRF1*, *KLRC1*, *XCL2*), erythroid (*HBB*, *HBD*), myeloid cells (*LYZ*, *S100A9*, *VCAN*), neutrophils (*LTF*, *LCN2*, *CAMP*), plasmacytoid DCs (pDC) (*IRF8*, *CLEC4C*), B cells (*CD79A*, *CD79B*), plasma cells (*CD79A*, *IGHG1*, *IGHA1*), and progenitor cells (*SPINK2*, *RUNX1*) (Figure 1C). Tumor cells were characterized by high expression of NB tumor signature genes (*PHOX2B*, *HAND2*, *STMN2*, and *KCNQ2*) (6), which were exclusively expressed in the BM of metastatic patients with NB (Figure 1D and Supplemental Figure 1, A and B). We also confirm the presence of tumor cells in NB bone metastatic samples using flow cytometry (Supplemental Figure 1C).

In addition, we performed inferCNV (23) analysis to confirm malignancy of tumor cells, showing notable copy number variations (CNVs) (Figure 1E). To identify the tumor cell state identity, we aligned metastatic BM data with primary NB scRNA-Seq data from previous studies (24). Metastatic tumor cells clustered with adrenergic cells and showed high expression of adrenergic signature genes *PHOX2B*, *MDK*, and *KCNQ2* (Figure 1, D and F). The presence of metastases significantly altered the immune cell composition of the BM. Notably, there was a trend toward an increased proportion of T cells and neutrophils and a trend toward a decreased proportion of B cells populations in the BM microenvironment of metastatic cases compared with nonmetastatic samples (Figure 1, B and G). Decreased B cell numbers were further confirmed by flow cytometry (Supplemental Figure 2, A and B).

### Analysis of myeloid cells identified tumor-associated neutrophils and macrophages involved in metastatic BM.

**Figure 2:**
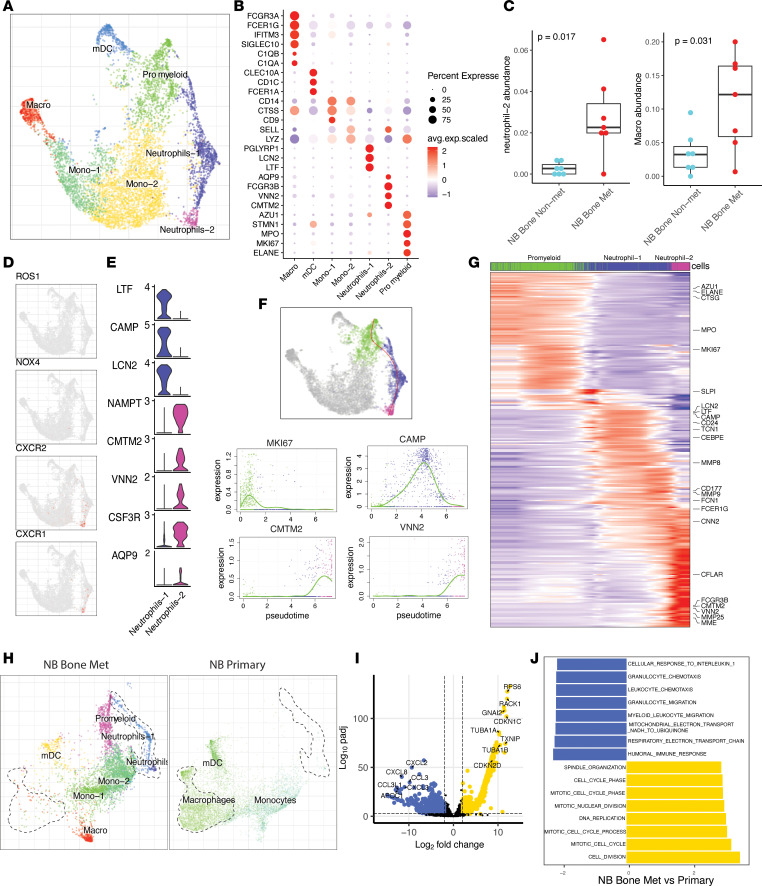
Myeloid cells characterization, enrichment, and differentiation trajectory. (**A**) Joint embedding represent the detailed annotation of myeloid cell subpopulations. (**B**) Dot plot demonstrating marker gene expression across different myeloid subpopulations. The color represents scaled average expression of marker genes in each cell type, and the size indicates the proportion of cells expressing marker genes. (**C**) Comparison of Neutrohil-1 and Neutrohil-2 proportion in NB bone metastatic (*n* = 7) and NB bone nonmetastatic (*n* = 7) samples. Statistics are accessed with 2-sided Wilcoxon ranked-sum test (*P < 0.05). For box plots, the center line represents the median, box limits represent upper and lower quartiles, and whiskers depicts 1.5 × the interquartile range (IQR). (**D**) UMAP embedding showing representative gene expression for neutrophiles. (**E**) Violin plot showing representative marker gene expression for 2 neutrophil subpopulations. (**F**) Estimated trajectory tree moving from promyeloid cells to Neutrophil-1 and Neutrophil-2 (top). Trajectory analysis demonstrates MKI67, CAMP, VNN2, and CMTM2 expression across pseudotime (bottom). (**G**) Heatmap showing the gene expression dynamics with pseudotime. Representative genes are shown for each cellular state along the cell differentiation. (**H**) Joint alignment of myeloid cells from NB bone metastatic tumor and NB primary tumor, visualized in UMAP embedding. (**I**) DEGs of macrophage comparing NB bone metastatic tumor with NB primary tumor, shown as volcano plot. The vertical dashed lines show the cut-off for gene filtering (log_2_FC 2 and −2), and the horizontal dashed lines signify adjusted *P* values of 0.01. (**J**) Enriched GO terms for DEGs from **I**. The statistical analysis was done using a hypergeometric test.

Since myeloid cells have been shown to play an important role in the tumor progression and metastasis (15, 25), we performed a detailed analysis of the myeloid cell composition. Focused analysis of the myeloid cell compartment revealed 7 major clusters: macrophages (*C1QA*, *C1QB IFITM3*, *SIGLEC10*), myelocytic DCs (mDC) (*CLEC10A*, *CD1C*, *FCER1A*), monocyte populations (Mono-1: *CD9*, *CTSS*; Mono-2: *SELL*, *LYZ*; ref. 26), progenitor myeloid cells (*MPO*, *MKI67*, *ELANE*, *AZU1*), and neutrophil populations (Neutrophil-1: *PGLYRP1*, *LCN2*, *LTF*; Neutrophil-2: *AQP9*, *FCGR3B*, *VNN2*, *CMTM2*) (Figure 2, A and B). Notably, the proportion of neutrophils increased in the metastatic samples (Figure 2C and Supplemental Figure 3A), transcriptionally resembling tumor-associated neutrophils (TAN) (27–29), with a high expression of *VEGFA*, *LGALS3*, *OLR1*, *PROK2*, *MMP9*, and *IL1RN* (Supplemental Figure 3, B and C). VEGFA are known to be proangiogenic factors. *MMP9* has been shown to play a crucial role in the angiogenic switch during tumor progression (30). Tumor-infiltrating neutrophils in colorectal cancer highly expressed *PROK2*, which is also involved in promoting angiogenesis (31). *OLR1* is associated with oxidized low-density lipoprotein, which has been described as a marker for distinguishing polymorphonuclear MDSCs (PMN-MDSCs) (32). Based on this observation, it is likely that TAN play a significant role in promoting angiogenesis and may contribute to tumor progression and metastasis by releasing proangiogenic factors and modulating inflammatory responses in the tumor microenvironment.

Our analysis revealed 2 TAN subtypes in the metastatic BM microenvironment. Recent high-resolution single-cell studies demonstrated diversity and plasticity of tissue-resident neutrophils in non–small cell lung cancer and liver cancer (27, 28). Neutrophils can inhibit tumor growth through release of reactive oxygen species (ROS) and reactive nitrogen species (RNS) (33). The expression of these molecules was not detected in the TAN from our data (Figure 2D), suggesting impaired antitumor activity. The Neutrophil-1 population expressed *LCN2*, *LTF*, *CAMP*, and *MMP8*. Neutrophil-2 was marked by high expression of *CSF3R*, *CMTM2*, *VNN2*, *AQP9*, *MMP9*, *MMP25*, *CXCR1*, and *CXCR2* (Figure 2E and Supplemental Figure 3C). As one of the inflammatory immune cell subtypes, neutrophils express chemokine receptors *CXCR1* and *CXCR2* (Figure 2D). Neutrophils expressing these molecules are attracted by chemokines produced by tumor cells that infiltrate the tumor microenvironment to form tumor-promoting activity (34, 35). Therapeutic strategies to target the CXCR-1/CXCR-2 axis or combination with immunotherapy have been proposed to improve antitumor efficacy in pancreatic cancer, metastatic melanoma, and metastatic colorectal carcinoma (35, 36). In addition, TAN can promote angiogenesis and dissemination of tumor cells by producing matrix metalloproteinases like *MMP9* and *MMP25* (37, 38), which were expressed in Neutrophil-2 (Supplemental Figure 3C).

Neutrophils originate from granulocyte progenitors (GMPs) in the BM (39). Therefore, we sought to dissect potential cellular trajectories for neutrophils. Our scRNA-Seq data reveal the hierarchical connection of neutrophil and myeloid progenitors, which suggests a trajectory from common myeloid progenitors toward Neutrophil-2 cell populations in metastatic BM (Figure 2F). Analysis of a transcriptional profile shows a continuous gene expression program associated with pseudotime (Figure 2G). Genes related to GMPs such as *MPO*, *ELANE*, and *AZU1* (39) and proliferating marker *MKI67* started to be expressed in the progenitor myeloid cells (Figure 2, F and G). Neutrophil-1 was marked by peak expression of granule genes such as *CAMP*, *LCN2*, and *LTF* previously tied to differentiating neutrophils (40). Neutrophil-2 are mature neutrophils labeled by high expression of *CXCR2* and *VNN2* (39) (Figure 2G and Supplemental Figure 3D). Notably, genes related to neutrophil migration and survival such as *NAMPT* (41), *CMTM2* (42), and *CD177* (43) showed increased gene expression and peak in Neutrophil-2 (Figure 2G and Supplemental Figure 3D). In summary, our in silico analyses demonstrate that neutrophils exhibit continuous trajectory within the metastatic BM microenvironment in NB.

The macrophage proportion also significantly increased in the metastatic samples compared with the nonmetastatic samples. On the contrary, the Mono-2 proportion decreased in the metastatic compartment (Supplemental Figure 3A). Our previous study on the primary NB immune microenvironment defined heterogenous subpopulations of tumor-associated macrophages (TAM) and monocytes (15, 24). We compared metastatic tumor myeloid cell compartments with localized primary NB tumors. Surprisingly, we found distinguished myeloid lineages in the primary and metastatic NB microenvironment. Myeloid progenitors and neutrophils were only present in the metastatic BM, while macrophages were predominantly enriched in the primary NB tumors (Figure 2H). Additionally, macrophages showed different phenotypes, where macrophages in primary NB tumors transcriptionally resembled tissue-resident macrophages, with high expression of markers such as *TREM2*, *FOLR2*, and *CD163* (44, 45). *TREM2*, *FOLR2*, and *CD163* are all markers found on tumor-associated M2 macrophages in adult cancer and have been shown to suppress the immune response and promote tumor growth (44–46) (Supplemental Figure 3E). In contrast, upregulated genes in BM-derived macrophages from metastatic BM were related to cell cycle and cell division (*CDKN1C*, *CDKN2D*, *RAC1*, *TUBA1A*) (Figure 2, I and J). These results suggest that different macrophage subtypes are in the site of the primary tumor compared with the BM of metastatic patients with NB. Overall, single-cell analysis of myeloid lineage identified increased numbers of neutrophils, with a TAN phenotype that activates genes involved in BM TAN differentiation and maturation as well as macrophage abundance in the metastatic BM microenvironment.

### Increased abundance of tumor-infiltrating CTLs and Tregs contributes to an immunosuppressive microenvironment.

**Figure 3:**
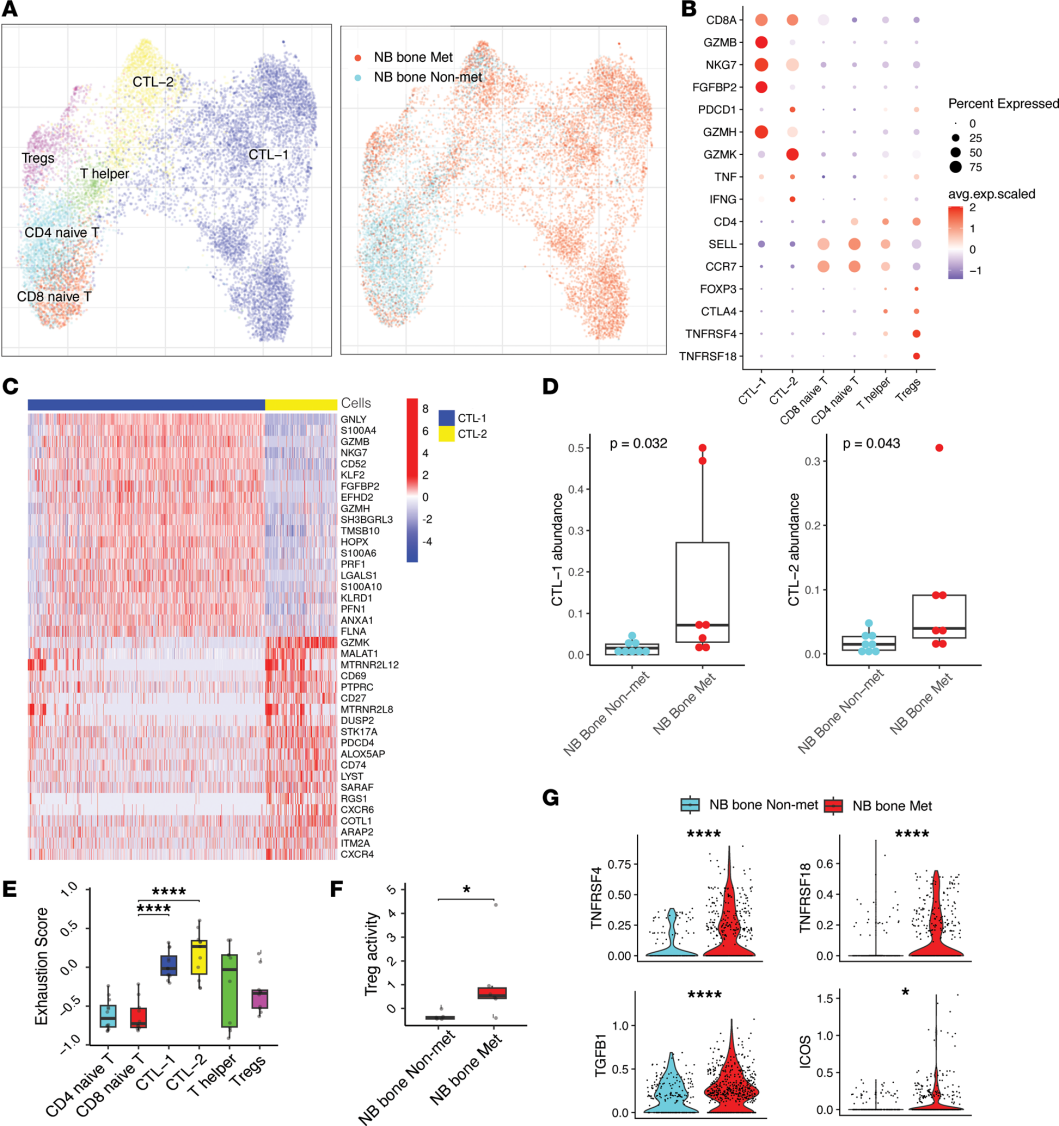
Increased abundance of tumor-infiltrating CTLs and Tregs in NB bone metastasis tumor. (**A**) UMAP embedding demonstrating T cell subpopulations (left) and sample fraction (right). (**B**) Dot plot demonstrating marker gene expression across different T cell populations. The color represents scaled average expression of marker genes in each cell type, and the size indicates the proportion of cells expressing marker genes. (**C**) Heatmap showing DEG in CTL-1 and CTL-2. (**D**) Comparison of CTL-1 and CTL-2 abundance in NB bone metastatic (*n* = 7) and NB bone nonmetastatic (*n* = 6) samples. Statistics are accessed with 2-sided Wilcoxon ranked-sum test (*P < 0.05). (**E**) Box plots showing T cell exhaustion score among different T cell subpopulations. Statistics are accessed with Wilcoxon ranked-sum test and Benjamini-Hochberg multiple-comparison correction (*P < 0.05). (**F**) Box plots illustrate significant increase of Treg activity in the metastatic tumor (NB bone metastatic, *n* = 7; nonmetastatic, *n* = 7). Statistics are accessed using 2-sided Wilcoxon ranked-sum test (*P < 0.05). For box plots (**D**–**F**), the center line represents the median, box limits represent upper and lower quartiles, and whiskers depicts 1.5 × the interquartile range (IQR). (**G**) Violin plots showing scaled log-normalized expression values of key genes in Tregs. A 2-sided Wilcoxon ranked-sum test was used for statistical analysis.

The BM is a primary reservoir site for immature T cells (19). To investigate T cell populations in metastatic BM patient samples, we performed unsupervised clustering on T cells and identified 6 distinct populations (Figure 3A), including 2 types of CTLs (CTL-1 and CTL-2; *CD8A*, *GZMB*, *PRF1*, *GZMK*), Tregs identified by the expression of *FOXP3* and *IL2RA*, naive T cells marked by *SELL* and *CCR7*, and Th cells identified by *CD4* and *CCR7*. CTL-1 and CTL-2 expressed different cytotoxic markers; CTL-1 showed high expression of *GZMB*, *GZMH* and *PRF1*, whereas CTL-2 was featured by *GZMK*, *CXCR6*, and *CD27*, indicating that these have a tissue resident memory phenotype (Figure 3, B and C). Both the CTL-1 and CTL-2 proportion increased in tumor metastatic niche, implying recruitment of T cells into the tumor metastatic microenvironment (Figure 3D). CTLs are known to target tumor cells via T cell receptor interaction with MHC class I and kill tumor cells through induction of apoptosis (47). However, in tumors, exhausted T cells have a decreased capacity to successfully eliminate tumor cells. To further analyze CTL cell states, we evaluated the T cell exhaustion gene signature score, where we detected a significantly increased level of expression in CTLs compared with other T cell subtypes, which is to be expected (19, 48) (Figure 3E and Supplemental Figure 4, A and B). We also evaluated individual genes playing important roles in exhaustion, comparing nonmetastatic and metastatic tumor CTLs. We showed significantly increased expression of *TOX*, *NR4A3*, *LAG3*, and *EOMES* in CTL-1 derived from metastatic samples, whereas CTL-2 from metastatic samples had significantly higher expression of all 3 *NR4A* genes tested in addition to *HAVCR2* and *LAG3* (Supplemental Figure 4C). Moreover, we performed flow cytometric analysis on 4 NB BM samples from the same patient cohort analyzed by scRNA-Seq and showed increased number of CD8^+^PD-1^+^ T cells in the metastatic sample compared with the nonmetastatic sample (Supplemental Figure 4, E and F). The upregulation of PD-1 alone is no conclusive indicator of T cell exhaustion, and we had a limited number of available patient samples. Therefore, we can conclude that we detected CTLs with increased expression of exhaustion-related genes in the metastatic BM niche compared with those derived from nonmetastatic BM. Additional functional analysis is needed to provide conclusive evidence regarding exhaustion in CTLs in the metastatic NB BM.

Besides CTLs, we also observed a trend of decreased naive T cells and increased Tregs in the metastatic BM (Supplemental Figure 4D). Studies have shown that high levels of Tregs can support the growth of metastatic tumors (49), a finding consistent with our data demonstrating a significantly increased Treg activity signature score in metastatic Tregs compared with Tregs from nonmetastatic BM (Figure 3F). Furthermore, differential gene expression analysis showed upregulation of *TNFRSF4*, *TNFRSF18*, *ICOS*, and *TGFB1* in metastatic BM compared with nonmetastatic BM (Figure 3G). TGFB1 is a cytokine that is produced by Tregs and plays a key role in the suppression of immune responses (50). Costimulatory molecules *ICOS*, *TNFRSF4*, and *TNFRSF18* are expressed on the surface of tumor-infiltrated Tregs and favor suppressive function of Tregs (51, 52). Increased expression of these molecules is known to result in increased activation and proliferation of Tregs, which could lead to a more effective suppression of tumor-associated immune responses, thus possibly contributing to tumor progression and metastasis (52, 53). Collectively, our data reveal an increase in tumor-infiltrating CTLs and Tregs that contributed to an immunosuppressive metastatic BM microenvironment in patients with NB.

### Unveiling the heterogeneity of NK and B cell subpopulations in metastatic BM.

**Figure 4:**
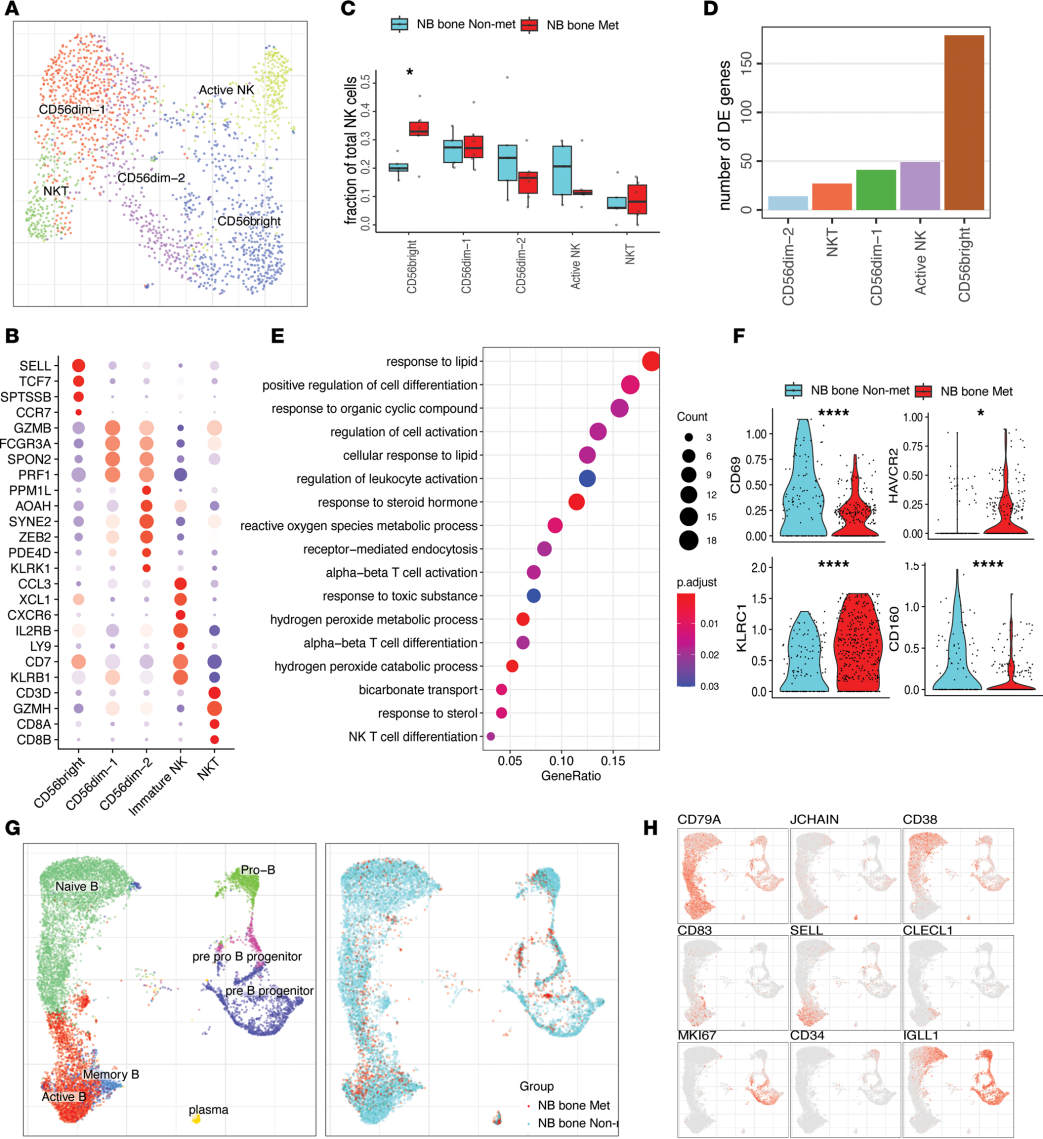
Characterization of B and NK cell subpopulations. (**A**) UMAP embedding of NK cells, color-coded by the cell subtypes. (**B**) Dot plot signifying marker gene expression across different NK cell subpopulations. The color represents scaled average expression of marker genes in each cell type, and the size indicates the proportion of cells expressing marker genes. (**C**) Box plot illustrating proportion of NK cell subpopulations in NB bone metastatic (*n* = 6) and NB bone nonmetastatic (*n* = 5) samples. Statistics significances are accessed using a 2-sided Wilcoxon ranked-sum test. For box plot, the center line represents the median, box limits represent upper and lower quartiles, and whiskers depicts 1.5 × the interquartile range (IQR). (**D**) Bar plot showing number of DEGs (adjust *P* < 0.05) for each NK cell subpopulation comparing NB bone metastatic and NB bone nonmetastatic samples. (**E**) Gene ontology showing the biological processes enriched in top 200 downregulated genes of CD56^bright^ NK cells comparing NB bone metastatic with NB bone nonmetastatic tumor. The color represents scaled average proportion marker genes in each cell, and the size indicates the number of CD56^bright^ cells. (**F**) Violin plots showing scaled log-normalized expression values of key genes in CD56^bright^ cell. A 2-sided Wilcoxon ranked-sum test was used for statistical analysis (*P < 0.05). ****P < 0.0001. (**G**) UMAP embedding demonstrating B cell subpopulations (left) and sample fraction (right). (**H**) Expression of key marker genes for B cell subpopulations.

A characterization of NK and B cell subpopulations within the metastatic BM microenvironment could increase our understanding of the role of these cells in modulating the immune response and potentially in improving the response to immunotherapy in these patients. Clustering of NK cells revealed 5 subpopulations (Figure 4A and Supplemental Figure 5A). NKT cells showed high expression of *NKG7*, *NCAM1*, *TBX21*, *CD8A*, *CD8B*, and *GZMH*. Active NK cells were marked by *CD7*, *CXCR4*, *IL2RB*, *CD69*, and *KLRB1*. We found 2 subpopulations of CD56^dim^ NK cells, CD56^dim-1^ (*FCGR3A*, *SPON2*) and CD56^dim-2^ (*FCGR3A*, *KLRK1*, *ZEB2*) (Figure 4B and Supplemental Figure 5A). Further analysis revealed that CD56^dim-2^ represented a terminally differentiated cell state with high expression of *HAVCR2* and *ZEB2* (54). CD56^dim-2^ also show high expression of *KLRK1*, which is one of the main NK cells activating receptor involved in antitumor activity (55). We observed a decrease of CD56^dim-2^ cell abundance in the metastatic BM. We also found that the CD56^bright^ (*CD56*, *TCF7*, *CCR7* and *SELL*) population was significantly increased in the metastatic BM (Figure 4C). This was accompanied with a significant change in differentially expressed genes (DEGs) in the CD56^bright^ population (Figure 4D and Supplemental Figure 5B). Downregulated genes were enriched for functions related to leukocyte activation, NKT cell differentiation, and α-β T cell activation (Figure 4E). Specifically, NK cell activating and stimulatory receptors *CD160* and *CD69* (56, 57) were significantly decreased in expression in metastatic BM compared with nonmetastatic CD56^bright^ cells, while the inhibitory receptor *KLRC1* (58) expression significantly increased in metastatic BM (Figure 4F). These findings suggest that the function of NK CD56^bright^ cells in the tumor BM metastatic environment is suppressed.

B cell development takes place in the BM. To investigate the B cell subtypes in our data set, we reclustered B cells to reveal 7 subpopulations: active B cells (*CD79A*, *MS4A1*, *BANK1*), memory B cells (*CLECL1*, *ZBTB32*), naive B cells (*CD38*, *IGLL1*, *CD69*), pre–B progenitors (*CD79A*, *MKI67*, *TOP2A*), pre/pro–B progenitors (*IL7R*, *CD99*, *SOX4*, *LEF1*), pro–B progenitors (*VPREB3*, *GNG11*, *PECAM1*, *RAG1*, *RAG2*, *DNTT*), and plasma cells (*JCHAIN*, *IGHG3*, *IGHG1*, *CD27*) (59, 60) (Figure 4, G and H, and Supplemental Figure 5C). Compared with nonmetastatic BM, the metastatic BM microenvironment showed a significantly decreased proportion of B cells, by scRNA-Seq and flow cytometry, possibly impairing B cell–mediated antitumor responses in patients with NB with metastatic BM (Figure 4G, and Supplemental Figure 2, A and B).

### A transcriptional metastatic NB signature predicts patient survival.

**Figure 5:**
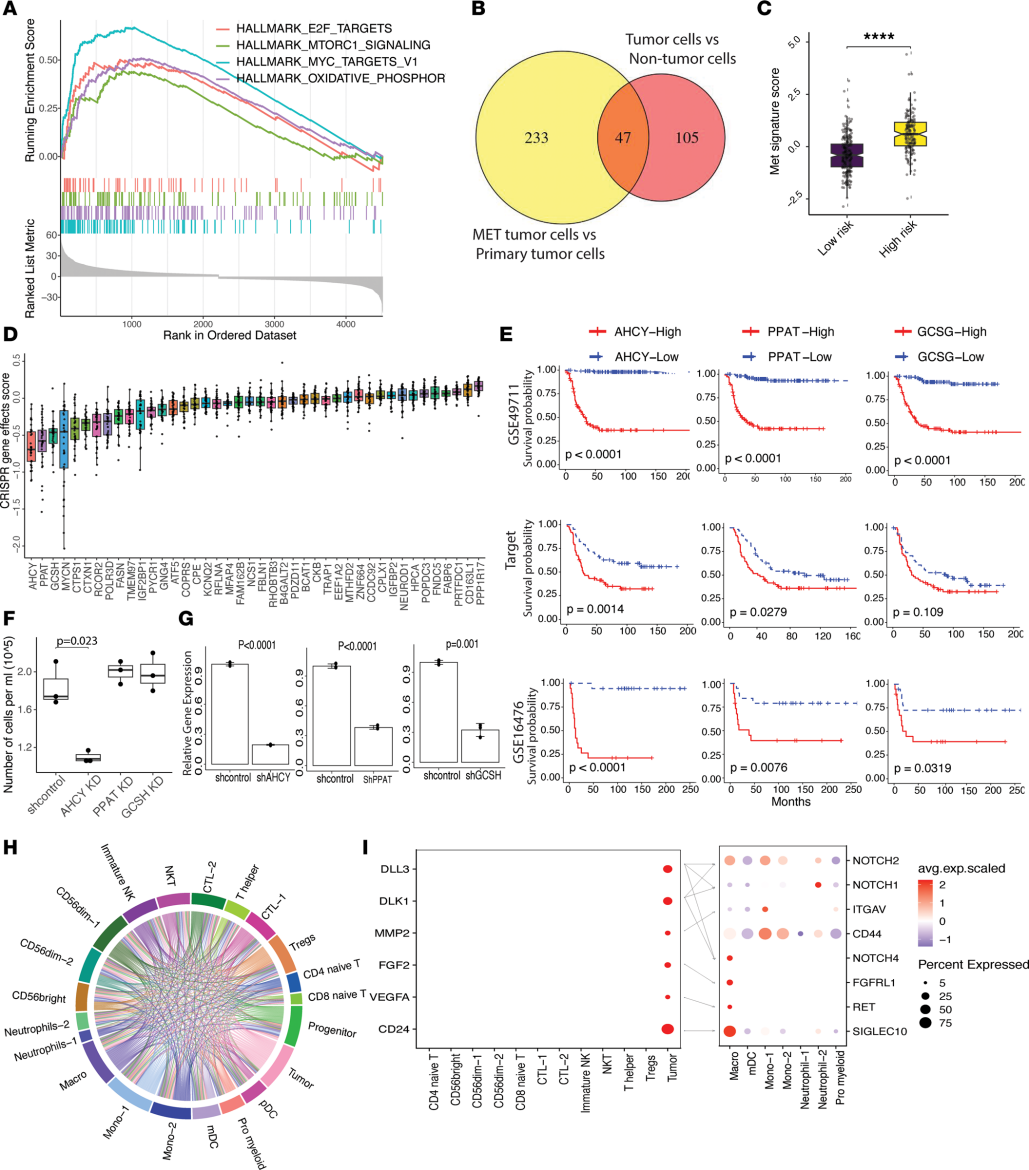
Metastatic signature predicts neuroblastoma patient overall survival. (**A**) Gene set enrichment (GSEA) plot depicting the enrichment pathways of genes upregulated in bone metastatic tumor cells against to primary NB tumor cells. (**B**) A Venn diagram illustrating the overlap of upregulated genes in bone metastatic tumor cells compared with non-malignant cells and primary NB tumor cells (see method). (**C**) Boxplot representing metastatic signature score in low-risk (*n* = 273) and high-risk (*n* = 172) patients with NB (GSE49711). Significance was assessed using 2-sided Wilcoxon ranked-sum test (***P < 0.001). (**D**) Boxplot showing the CRIPSR screen effect score of metastatic signature genes in neuroblastoma cell lines (*n* = 35). Effect Scores indicate whether gene knockout (gene loss) has a positive or negative effect on the growth or survival of cancer cells. (**E**) KM survival curves showing patients with NB with higher metastatic signature gene expression have worse overall survival in 3 independent NB data sets (GSE49711 *n* = 488, Target *n* = 247, GSE16476 *n* = 76). Patients were stratified into 2 groups based on the gene expression (binary: top 25% versus bottom 25%). Statistics are accessed by 2-side log-rank test. (**F**) Boxplot showing cell growth of neuroblastoma cell line (TET21N) on day3 after shRNA infection (*n* = 3). (**G**) Barplot showing relative mRNA expression (*n* = 3). Data are expressed using the 2−ΔΔCt method. Gene expression levels were normalized to the sh control. Statistical significance determined using 2-sided t-test. (**H**) Overview of potential ligand-receptor interactions of cell subpopulations. (**I**) Dot plot showing significant ligand (tumor cells and T cell subsets) and receptor (myeloid cell subsets) expression. Dot size indicates expression ratio, color represents average gene expression (Methods). Boxplots include center line, median; box limits, upper and lower quartiles; whiskers are highest and lowest values no greater than 1.5× IQR. ****P < 0.0001.

During metastasis, cancer cells disseminate from the primary tumor spread and colonize distant organs. We compared transcriptomic alterations of primary NB tumor cells to bone metastatic tumor cells with the hypothesis that a gene signature may be indicative of tumor progression and metastasis. Differential gene expression analysis revealed upregulated genes in metastatic BM tumor cells related to E2F targets, oxidative phosphorylation, and mTORC1 signaling as well as MYC targets (Figure 5A). mTORC1 is a major regulator of cell proliferation and motility and, thus, can play a role in the invasion and metastasis of tumors (61). Oxidative phosphorylation is one of the major pathways for ATP production. Tumor cells rely on oxidative phosphorylation to obtain the energy they need to migrate, invade, and establish new colonies in distant tissues (62). MYC and E2F activation can lead to increased tumor cell proliferation, migration, and invasion, which can contribute to tumor metastasis (63, 64).

By filtering DEGs from nontumor cells and primary tumor cells, we restricted those DEGs specific to metastatic tumor cells. In total, we identified 47 overlapping genes that were significantly upregulated in the metastatic tumor cells (Figure 5B and Supplemental Figure 6, A and B). To test if the gene signature is indicative of tumor progression and disease stage, these gene signatures were then applied to publicly available NB bulk RNA-Seq data. The average metastasis scores were calculated from the bulk RNA-Seq data as described in Methods. The metastatic signature score was significantly increased in patients with high-risk and stage 4 NB (Figure 5C and Supplemental Figure 6C), and a higher expression of the metastatic signature score was associated with worse overall survival outcomes in 3 independent NB cohorts (Supplemental Figure 6D).

We next used a publicly available CRISPR Screen data set (DepMap) (65) on NB cell lines to narrow the list of genes that could predict survival outcome. Notably, we observed that *AHCY*, *PPAT*, and *GCSH* show a lower effect score closed to *MYCN* (Figure 5D), suggesting that the KO or suppression of those genes have a negative effect on the growth or survival of NB cell lines. *MYCN* amplification is one of the strongest predictors of poor prognosis in patients with NB (66). We hypothesized that *AHCY*, *PPAT*, and *GCSH* could, therefore, also be prognostic biomarkers. Survival analysis show that upregulation of *AHCY*, *PPAT*, and *GCSH* alone are significantly associated with worse patient survival in 3 independent NB patient cohorts (Figure 5E). These genes could distinguish primary tumors from metastatic tumors, suggesting that they are involved in the metastatic process and may, therefore, predict tumors with a high likelihood to metastasize to the BM. Next, we therefore explored the therapeutic effect of these genes in a *MYCN* amplified NB cell line (TET21N). Here, we found that knockdown of the *AHCY* gene by short hairpin RNA (shRNA) significantly impaired the growth of NB cells in vitro (Figure 5, F and G). The *AHCY* gene codes for the enzyme S-Adenosyl-L-homocysteine hydrolase. Knocking down this gene has been shown to lead to DNA damage and cell cycle arrest (67, 68), suggesting that the knockdown effects observed in NB cell survival might be due to this mechanism. Targeting AHCY could be a therapeutic approach for patients with high-risk NB and warrants further studies.

Additionally, we performed cell-to-cell interaction analysis to explore how metastatic tumor cells might interact with other cells within the metastatic BM microenvironment (Figure 5H). Ligand-receptor analysis uncovered several significant interaction channels, including ligands that were distinctively upregulated in the tumor cells such as *CD24*, *VEGFA*, *DLL3*, and *DLK1* (Figure 5I and Supplemental Figure 6E). Interestingly, tumor cells expressing CD24 may promote immune evasion through its interaction with the inhibitory receptor SIGLEC10 expressed by macrophages (69). Our data show that *FGFRL1* is expressed in macrophages, and FGF2 has been shown to regulate programming of TAM and to control tumor growth and antitumor immunity (70). We also observed an increase in Notch signaling pathway receptors *NOTCH1* and *NOTCH2* expressed in Neutrophil-2, while *NOTCH2* and *NOTCH4* were expressed in macrophages. The Notch signaling pathway has been shown to regulate various aspects of the tumor immune response, such as immune differentiation and maturation, as well as recruitment of neutrophils and regulation of immunosuppressive TAM (36). Furthermore, our data demonstrate high expression of Notch ligands (*DLL3*, *DLK1*) in tumor cells, indicating a possible role for these ligands in the recruitment and regulation of TAN and TAM within the bone metastatic microenvironment. Taken together, this analysis uncovers potential communication channels between tumor cells and the surrounding microenvironment and reveals a metastatic signature that was significantly associated with worse patient survival.

## Discussion

NB is the most common and deadliest tumor of infancy, and BM metastasis is linked to poor prognosis with limited therapy options (5). In this study, single-cell transcriptomics was used to characterize the microenvironment of metastatic BM in patients with NB. We provide a single-cell landscape of tumor cells and immune cells within the BM tumor microenvironment. Several distinct cell types were enriched in metastatic BM, forming an immune-suppressive microenvironment with a composition of TAN, Tregs, and exhausted T cells. These immune cells showed dysregulated transcriptional profiles and upregulation of pathways that are associated with suppressing the antitumor immune response and might, therefore, contribute to metastatic growth in the BM. Our findings provide insight into the complex microenvironment of metastatic BM in human NB.

Immune suppression within the BM seems to be a key feature in our patient cohort. Immune suppression can be caused by various factors, including tumor-secreted cytokines and chemokines, increased expression of immune checkpoint molecules, alterations in NK cell function, and infiltration of suppressive immune cells such as TAM and Tregs (12, 71). TAM are known to produce proteolytic enzymes that degrade the bone matrix, allowing the tumor cells to penetrate and migrate to the bone (72). Within the myeloid lineage, we demonstrated an increase in immune-suppressive myeloid cells such as TAM and TAN in metastatic BM samples. This finding is in line with our previous work on adult prostate cancer demonstrating increased TAM in the bone metastatic niche (21).

Of note, we identified 2 TAN subpopulations exclusively enriched in metastatic NB BM. In tumors, neutrophils are often found at the tumor site and are thought to promote the growth and spread of tumors by providing a favorable environment for tumor cells (73). Our data show that neutrophils exhibited suppression of antitumor immune responses and produced matrix metalloproteinases that can act to promote angiogenesis and invasion. In addition, genes related to neutrophil migration and survival — such as *NAMPT*, *CMTM2*, and *CD177* — were also increased in expression in Neutrophil-2. Thus, we hypothesize the TAN infiltration might play an important role in dissemination and immune cell escape of metastatic tumor cells in the BM of patients with NB. Neutrophil recruitment to the tumor microenvironment is mediated by multiple mediators, including cytokines, chemokines, and growth factors. We observed increased expression of receptors such as *CXCR2*, *CXCR1*, and *CSF3R* in TAN that have been reported to be crucial for neutrophil recruitment to the tumor parenchyma (34, 35, 74). In addition, expression trajectory analysis of TAN development indicated continuous TAN lineage trajectory within the metastatic BM microenvironment, suggesting that these TAN are modified by tumor-mediated signals to shape them into immunosuppressive neutrophils. Future orthogonal validation experiments are needed to provide conclusive evidence regarding the role of TAN in the metastatic NB BM.

Besides TAM and TAN, we also observed an increase in exhausted CTLs and dysregulated NK cells in the metastatic BM. T and NK cell gene expression patters showed increased expression of immune checkpoint molecules and decreased expression of cell activation molecules. Immunotherapy has been shown to be effective in treating a number of different cancer types, including melanoma, lung cancer, and bladder cancer (75). Our data demonstrate that the metastatic BM in NB have accumulated CTLs, suggesting a potential target modulation of these cells to improve immunotherapy in patients with bone metastatic NB.

The high degree of NB tumor cell heterogeneity has been the topic of considerable research focus (6, 9). NB tumors comprise diverse populations of cells with different genetic backgrounds; these cell populations affect progression, metastatic potential, and treatment response. Due to the limited number of patients and tumor cells, we cannot comment on tumor cell heterogeneity. However, a comparison of metastatic BM tumor cells with primary localized tumor cells identified a set of upregulated genes, suggesting that these genes may be involved in metastatic progression. These also defined a metastatic signature that could accurately predict overall survival in patients with NB.

Although our analysis presents a good representation of immune and tumor cells in the BM metastatic niche, it is important to consider a few potential limitations to our study. One of the main limitations is lack of validation. Although we performed functional interpretation and protein validation of certain cell types, validation in more patient samples will be necessary to further substantiate these findings. CRISPR Screen data reveal varying effect scores in NB cell lines; it is important to validate these results in a wider range of cell lines to fully understand the therapeutic implications. In addition, our analysis can’t determine the cause-and-effect relationship of TME remodeling. Metastasis is a dynamic process that spans multiple organs and occurs over extended periods of time (76, 77). It is challenging to discern a definitive cause-and-effect relationship in our current experiment design. Furthermore, cell-to-cell interaction analysis was built on single-cell data, and it is crucial to perform spatial transcriptomic data analysis to understand how immune cells infiltrate tumors and to identify targets that can modulate the interaction between immune cells and tumor cells in the metastatic BM microenvironment. Nevertheless, in spite of these difficulties, we managed to uncover notable disparities that could have a profound clinical implication in discerning the effect of immune and tumor cells on the survival of patients with metastatic NB.

Overall, our study provides an important step forward in understanding the complexity of the tumor microenvironment, specifically in the metastatic BM in patients with NB. We believe that our systems biology approach to understand metastatic NB provides a rich resource for the further study of metastatic disease.

## Methods

### Sex as a biological variant.

The participants in our study were randomly selected, and it included both male and female patients with NB. Sex was not considered as a biological variable.

### Surgical approach and collection of tumor and BM specimens.

BM aspirates were collected from 8 patients diagnosed with metastatic NB and 7 patients with nonmetastatic disease. Bone tumor tissue was surgically resected from the bone. All patients consented to having their BM and tissue used for research purposes.

Samples were taken from each patient while they were in the prone position under general anesthesia, as their spine was approached from the back.

### BM processing.

BM samples were filtered through a 70 μm filter and were then centrifuged at 600*g* for 7 minutes at 4°C. Plasma was collected, and erythrocytes were removed using ACK Lysing buffer (Quality Biological). The cells were resuspended in Media 199 supplemented with 2% (v/v) FBS for further analysis.

### Dissociation of tissues into single cells of bone tumor.

All samples were collected in Media 199 supplemented with 2% (v/v) FBS (Gibco, Thermo Fisher Scientific). Tumor pieces measuring 1 mm^3^ were cut using a 70 mm filter cap, followed by enzymatic dissociation at 37°C with shaking at 120 rpm for 45 minutes. The dissociation solution contained Collagenase I, II, III, and IV (all 1 mg/mL; Worthington) and Dispase (2 mg/mL; Gibco, Thermo Fisher Scientific) as well as RNase inhibitors (RNasin [Promega] and RNaseOUT [Gibco, Thermo Fisher Scientific]). Erythrocytes were removed using ACK Lysing buffer, and cells were then resuspended in Media 199 with 2% (v/v) FBS for further analysis.

### FACS of human samples for scRNA-Seq.

Single cells from tumor and BM were subjected to RBC lysis and then surface stained with anti–CD235-PE (BioLegend, catalog 306609) for 30 minutes at 4°C. Afterward, cells were washed with 2% FBS-PBS (v/v) and DAPI stained (1 μg/mL). Live and nonerythroid cells (DAPI^–^CD235^–^) were flow sorted on a BD FACS Aria III instrument with a 100 μm nozzle (BD Biosciences). Flow cytometry data were then analyzed using FlowJo software (Tree Star Inc.).

### Massively parallel scRNA-Seq.

Single cells were encapsulated into emulsion droplets using the Chromium Controller (10X Genomics). scRNA-Seq libraries were prepared using the Chromium Single Cell 3’ v2 Reagent Kit, following the manufacturer protocol. After sorting, sample volumes were reduced and cells were observed under a microscope and counted with a hemocytometer. Approximately 6,000 cells were then loaded into every channel. cDNA and library preparation was conducted on a C1000 Touch Thermal cycler with a 96-Deep Well Reaction Module (Bio-Rad). Amplified cDNA and final libraries were assessed using an Agilent BioAnalyzer and a High Sensitivity DNA Kit (Agilent Technologies). Individual libraries were diluted to 4 nM and combined for sequencing. The pools were sequenced with 75 cycle run kits (26 bp, read 1; 8 bp, index 1; and 55 bp, read 2) on the NextSeq 500 Sequencing System (Illumina), achieving an approximately 70%–80% saturation level.

### Processing and analyzing scRNA-Seq data.

Using Cell Ranger (v.3.0.1) software, the raw scRNA-Seq data were aligned to the human GRCh38 genome with default parameters, and low-quality cells with fewer than 600 total UMIs detected were filtered out. Subsequently, cells were further analyzed with Scrublet (78), and cells with a Scrublet score above 0.4 were excluded. In total, 37,406 cells from 15 samples were obtained (Supplemental Table 2). Conos (22) was then used (k = 15, k.self = 5, matching.method = ‘mNN’, metric = ‘angular’, space = ‘PCA’) to integrate the multiple scRNA-Seq data sets, and principal component analysis was performed on the 2,000 genes with the most variable expression. Leiden clustering was used to build joint cell clusters across the entire data set collection, and UMAP embedding was estimated using the embedGraph function in Conos with default parameter settings.

### Identifying the major cell types and cell subpopulations.

To identify the major cell types present in both the NB nonmetastatic and bone metastatic BM samples, we applied sets of well-established marker genes for each cell type to annotate the cell types based on the most highly expressed genes. The detailed gene list can be found in Supplemental Table 3. Furthermore, we used Conos to analyze cell subsets separately and identify any subclusters within the major cell types. Specifically, we extracted all myeloid cells (T cells/myeloid cells/B cells/NK cells), removed low-quality samples with less than 40 cells per cells, and realigned separately using Conos with default parameters.

### Calculation of gene set signature scores.

Calculation of Gene Set Signature Scores involves analyzing the expression levels of individual genes in a given set to determine the overall effect of a given gene set on a biological system. This is done by measuring the expression levels of each gene in the gene set and then combining them (average normalized gene expression) to create a score that reflects the overall effect of the gene set on the system. T cell exhaustion and Treg activity signature genes are listed in Supplemental Table 4. The statistical significance was assessed using Wilcoxon ranked-sum test.

### Differential gene expression analysis.

For differential expression analysis between cell types, the Wilcoxon ranked-sum test, implemented by the getDifferentialGenes() function from Conos, was used to identify marker genes of each cell cluster. Genes were considered differentially expressed if the *P* value–determined *Z* score was greater than 3. For differential expression analysis between sample fractions (e.g., nonmetastatic NK versus metastatic NK), the estimatePerCellTypeDE function in Cacoa (79) was utilized. This first formed “mini-bulk” (or meta-cell) RNA-Seq measurements by combining all molecules measured for each gene in each subpopulation in each sample. Subsequently, differential gene expression analysis was performed using DESeq2 (80) with default settings. A minimal number of 10 cells (of the selected cell type) was required for a sample to be included in the comparison.

### Defining metastatic gene signature.

To identify the malignant cells, we analyze DEGs. Malignant cells were marked by high expression of NB tumorigenesis genes (*PHOX2B*, *HAND2*, *STMN2*, and *KCNQ2*). Next, we inferred large-scale chromosomal CNVs with inferCNV, which uses a moving averaged expression profile across chromosomal intervals (23), comparing data with normal reference data. We considered immune cells as the reference cells. By comparing metastatic tumor cells with primary NB tumor cells, and requiring genes exclusively expressed in tumor cells, we identified a 47-gene set metastatic signature (Supplemental Table 5). getDifferentialGenes() function from Conos (22) was used to calculate DEGs, and *P* value–determined *Z* scores (cutoff of 10) were used to filter upregulated gene in metastatic tumor cells.

### Align metastatic BM data with primary NB scRNA-Seq data.

For the joint alignment analysis with primary NB data, we downloaded raw count matrix and cell annotation from GSE147766 (24). Conos was used to integrate multiple samples together with default parameter settings.

### Survival analysis.

To test if a given signature predicts survival, we first computed the average expression of the signature in each tumor based on the bulk RNA-Seq data. Next, we stratified the patients into 2 groups according to the average expression of the signature: high or low expression correspond to the top or bottom 25% of the population, respectively. We used log-rank test to examine if there was a significant difference between patient groups in terms of their survival. R package survival and survminer were used to draw Kaplan-Meier (KM) plot.

### Gene Ontology and GSEA.

The clusterProfiler R package (81) was used to test for enriched Gene Ontology (GO) Biological Processes or KEGG Pathways in gene sets, using default parameters. To identify the enriched Biological Processes GO Terms, the approach above was applied to the top 300 upregulated genes determined by the estimatePerCellTypeDE functions of Cacoa (79). For GSEA, we ranked genes by *Z* score, and GSEA function from clusterProfiler was used to test enriched cancer hallmark pathways.

### Trajectory analysis.

We use Slingshot (82) and crestree (83) to perform trajectory analysis of neutrophil development. Specifically, we extracted progenitor monocytes, Neutrophil-1 and Neutrophil-2 cells from myeloid lineage. The pseudotime was estimated using slingshot() function from Slingshot; then, we analyzed genes that were significantly associated with pseudotime using test.associated.genes() function from crestree.

### Ligand and receptor analysis.

We inferred ligand-receptor interactions using a method similar to that described in a previous study (84). From the CellDBphone database, we collected 1,263 well-annotated ligand-receptor pairs. We first screened each ligand and receptor based on their expression levels in each cell type, requiring that the gene be expressed in at least 10% of the cells. Subsequently, we calculated the average expression of ligand-receptor pairs across cell type pairs using normalized scRNA-Seq data. The product of the average expression of the ligand in cell type A and the average expression of the receptor in cell type B was used to measure the expression of the ligand-receptor pair. To evaluate the robustness and statistical significance of the ligand-receptor pairs, we constructed a null distribution for average ligand-receptor expression by shuffling cell identities in the aggregated data and then calculating the ligand-receptor average pair expression across 1,000 permutations of randomized cell identities. The *P* value was the number of randomized pairs that exceeded the observed data. To prioritize functional ligand-receptor interaction pairs in tumor tissue, we further conducted a differential gene expression analysis, requiring ligand exclusively expressed in tumor cells. getDifferentialGenes() function from Conos was used in differential gene expression analysis. The ligand-receptor list can be found in Supplemental Table 6.

### Flow cytometry analysis.

Flow cytometric validation was performed on nonmetastatic and NB metastatic BM samples that were matched with the scRNA-Seq data. Matched sampling enables the direct comparison and validation of scRNA-Seq data at a protein level, while controlling for interindividual variabilities. Cells were thawed at 37°C and washed with prewarmed media RPMI supplemented with 2% (v/v) FBS; blocking was performed with anti–human fc block (BD Pharmingen, 564219) for 10 minutes. Subsequently, cells were stained for surface markers with antibodies diluted 1:200 in 2% FBS-PBS (v/v) for 30 minutes at 4°C while protected from light, as indicated in the antibody panels provided in Supplemental Table 7. After the antibody incubation, cells were washed with 2% FBS-PBS (v/v), followed by live/dead fixable dead cell staining for 10 minutes; they were then washed with PBS. Cells were fixed and permeabilized for 30 minutes with Cytofix/Cytoperm before being washed with 1× Perm/Wash buffer (BD Biosciences). Unstained cells and FMOs served as negative controls, and UltraComp eBeads Plus Compensation Beads (Invitrogen, 01-3333-42) were used for compensation of each antibody. Data were acquired in Sony ID7000 Spectral Analyzer and analyzed with FlowJo software (v 10.9.0) and GraphPad Prism. Gating strategy is presented in Supplemental Figure 1D and Supplemental Figure 3E. For the tumor cell panel, harnessing the same tumor surface markers (CD276, GD2, CD24, CD56), we designed a flow cytometry staining panel to identify, detect, and quantify the proportion of tumor cells in metastatic BM samples — BM1 and BM3 — that were matched with the scRNA-Seq data.

Samples were thawed at 37°C and washed with prewarmed media RPMI supplemented with 2% (v/v) FBS; blocking was performed with anti–human fc block (BD Pharmingen, 564219) for 10 minutes. Subsequently, cells were stained for surface markers with antibodies diluted in 2% FBS-PBS (v/v) for 30 minutes at 4°C while protected from light, as indicated in the antibody panels provided in Supplemental Table 7. After the antibody incubation, cells were washed with 2% FBS-PBS (v/v), followed by DAPI staining, for subsequent data acquisition in BD FACSAria Fusion and analysis with FlowJo software (v 10.9.0).

### Quantitative PCR (qPCR).

Total RNA from TET21N cells was extracted using RNeasy Micro Kit (Qiagen, 74004). cDNA was synthesized from total RNA using iScript cDNA Synthesis Kit (Bio-Rad, 1708891). qPCR was performed using iTaq Universal SYBR Green Supermix (Bio-Rad, 1725121) on a CFX96 Real-Time System (Bio-Rad). The data were analyzed using the 2^–ΔΔCt^ method. ACTB was used as housekeeping genes. The following primers were used for qPCR analysis: ACTB, 5′-AGAGCTACGAGCTGCCTGAC-3′, 5′-AGCACTGTGTTGGCGTACAG-3′; AHCY, 5′-ATCCTCAAGGTGCCTGCCATCA-3′, 5′-CGGCAATCATCACATCTGTGGC-3′; PPAT, 5′-GCGATTGAAGCACCTGTGGATG-3′, 5′-CGGTTTTTACACAGCACCTCCAC-3′; and GCSH, 5′-GGCATTGGAACAGTGGGAATCAG-3′, 5′-CACACTTTCCAAAGCACCAAACTC-3′.

### Lentivirus for knockdown.

Lentiviral constructs were transfected with VSVG and Δ8.9 (packaging) to HEK293T cells using FuGENE6 (Promega, E2692). The viral supernatant was then concentrated using Lenti-X (TaKaRa, 631232) and transduced into recipient cells with 8 μg/mL polybrene. After transduction, the infected cells were selected with puromycin for 2 days. Knockdown experiments used pLKO-Tet-On vector (Addgene, 21915). In total, 1 μg/mL doxycycline (DOX) was used to induce knockdown. RNA was extracted on day 5 for qPCR (Supplemental Table 8). The following shRNAs sequences were used. shAHCY, 5′-CACAGGCTGTATTGACATCAT-3′; shPPAT, 5′-CAATACCATCTCACCTATAAT-3′; shGCSH, 5′-GTGAACTCTATTCTCCTTTAT-3′. Control (Renilla), 5′-TAGATAAGCATTATAATTCCT-3′.

### Statistics.

*P* < 0.05 was considered significant. Two-sided Wilcoxon ranked-sum test was used to assess significance in bulk RNA-Seq and scRNA-Seq analyses unless otherwise stated.

### Study approval.

Patient material and all patient tissue collection was carried out with IRB approval, Etikprövningsmyndigheten. Diary number (registration): 2009/1369-31/1 and 2022-07254-01.

### Data availability.

Raw sequencing data and processed data in this paper are available under the accession no. GSE220946. For the joint alignment analysis with primary NB data, we downloaded raw count matrix and cell annotation from GSE147766 (24) (https://www.ncbi.nlm.nih.gov/geo/query/acc.cgi?acc=GSE147766). Moreover, NB bulk RNA-Seq data were download from GSE16476 (85), GSE49711 (86), and cBioPortal (87). (https://cbioportal-datahub.s3.amazonaws.com/nbl_target_2018_pub.tar.gz). The codes generated during this study are available at GitHub repository (https://github.com/shenglinmei/NB.bone.Met) (commit ID: 27b633c). Values for all data points in graphs are reported in the Supporting Data Values file.

## Author contributions

Co-first authorship order was based on the timeline of contributions. Conceptualization was contributed by SM, AMA, PK, JIJ, DBS, PVK, and NB. Sample collection methodology and surgeries were contributed by AMA, BTE, PK, and JS. Investigation was contributed by SM, AMA, BTE, IMG, PVK, and NB. Computational investigation and qPCR analysis were contributed by TZ, XL, and NEJ. FACS analysis was contributed by AMA, BTE, AP, SM, TKO, ORB, PVK, and NB. Writing of the original draft was contributed by SM, AMA, and NB. Review and editing of the manuscript were contributed by SM, AMA, BTE, BMV, HS, PK, MW, ORB, JS, JIJ, PJS, DBS, and NB. All authors read, edited, and approved the manuscript. Funding acquisition, resources, and supervision were contributed by PK and NB.

## Supplementary Material

Supplemental data

Supplemental tables 1-8

Supporting data values

## Version 1 02/15/2024

In-Press Preview

## Version 2 03/22/2024

Electronic publication
