# Supplementary material for: Single-cell analyses of metastatic bone marrow in human neuroblastoma reveals microenvironmental remodeling and metastatic signature
Source: JCI Insight. 2024 Feb 15;9(6):e173337. doi: 10.1172/jci.insight.173337 (PMC10972621; doi:10.1172/jci.insight.173337)
Supplement: Supplemental data [file jciinsight-9-173337-s080.pdf]

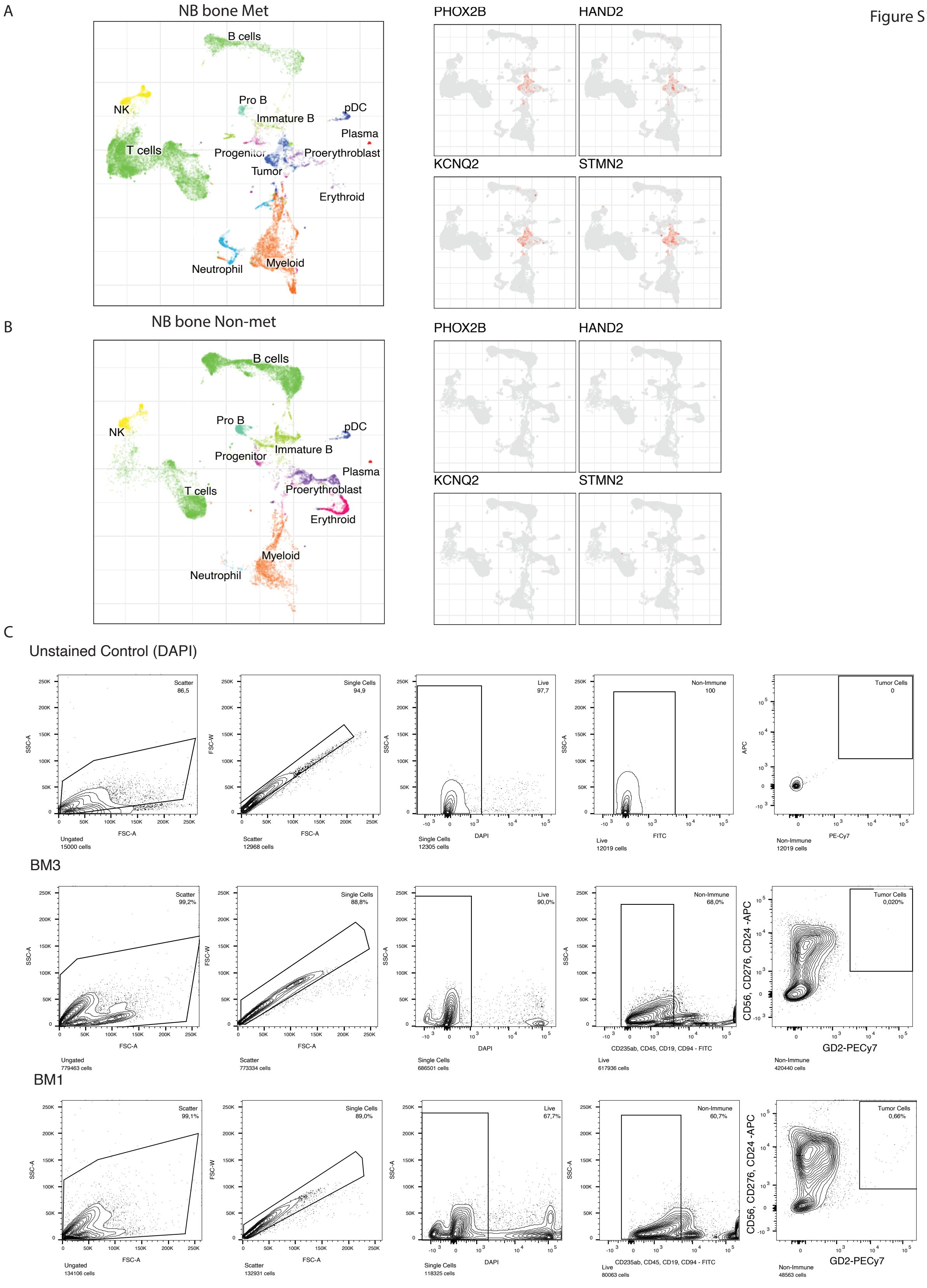

A
NB bone Met
B cells
NK
Pro B
Immature B
pDC
Plasma
T cells
Progenitor
Proerythroblast
Tumor
Erythroid
Neutrophil
Myeloid
PHOX2B
HAND2
KCNQ2
STMN2
B
NB bone Non-met
B cells
NK
Pro B
pDC
Progenitor
Immature B
Plasma
T cells
Proerythroblast
Erythroid
Myeloid
Neutrophil
PHOX2B
HAND2
KCNQ2
STMN2
C
Unstained Control (DAPI)
Scatter 86,5
Ungated 15000 cells
Single Cells 94,9
Scatter 12968 cells
Live 97,7
Single Cells 12305 cells
Non-Immune 100
Live 12019 cells
Tumor Cells 0
Non-Immune 12019 cells
SSC-A
FSC-A
FSC-W
DAPI
FITC
APC
PE-Cy7
BM3
Scatter 99,2%
Ungated 779463 cells
Single Cells 88,8%
Scatter 773334 cells
Live 90,0%
Single Cells 686501 cells
Non-Immune 68,0%
Live 617936 cells
CD235ab, CD45, CD19, CD94 - FITC
Tumor Cells 0,020%
Non-Immune 420440 cells
CD56, CD276, CD24 -APC
GD2-PECy7
BM1
Scatter 99,1%
Ungated 134106 cells
Single Cells 89,0%
Scatter 132931 cells
Live 67,7%
Single Cells 118325 cells
Non-Immune 60,7%
Live 80063 cells
CD235ab, CD45, CD19, CD94 - FITC
Tumor Cells 0,66%
Non-Immune 48563 cells
CD56, CD276, CD24 -APC
GD2-PECy7

Figure S1

Supplementary Figure 1. Overview of tumor microenvironment of neuroblastoma bone metastatic and non-metastatic tumors

A. UMAP visualization of joint alignment of NB bone Met samples, color-coded by the cell subtypes and representative tumor marker genes.

B. UMAP visualization of joint alignment of NB bone Non-met samples, color-coded by the cell subtypes and representative tumor marker genes.

C. Gating strategy for enrichment of *CD2*+ tumor cells in NB bone met samples BM1 and BM3.

A

Gating strategy CD19+ B cells

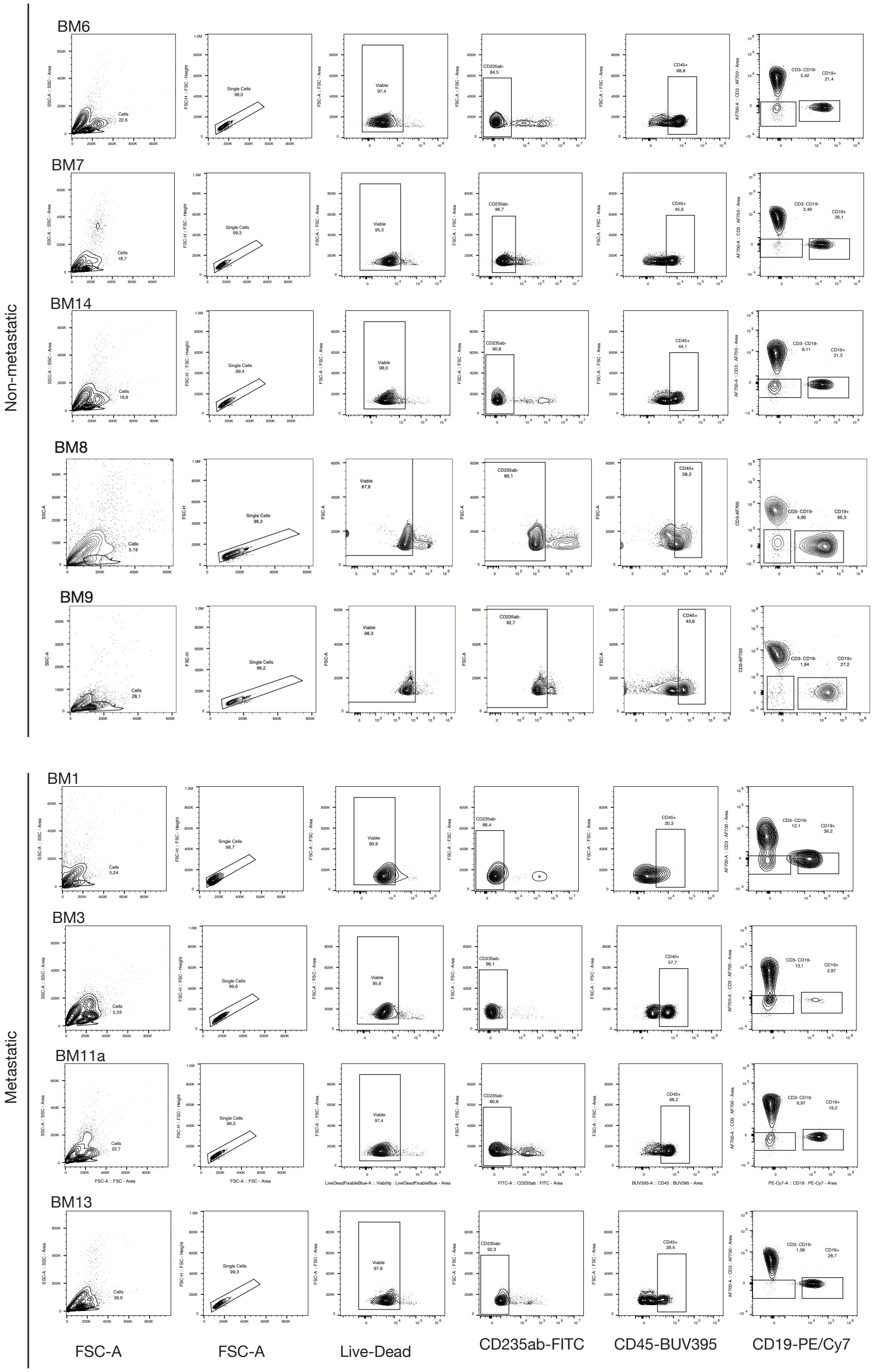

B

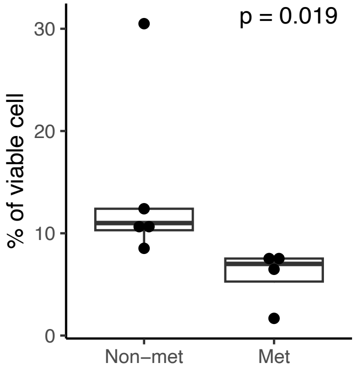

Supplementary Figure 2. Gating strategy for B cells

A. Gating strategy for enrichment of *CD19*+ B cells. Labels above the flow plots refer to the parent population in the percentages are of the parent gate.

B. Boxplot showing the percent of *CD19*+ B cells in NB bone Met and NB bone Non-met samples (n=3). Statistical significance determined using two-sided t-test. Boxplots include center line, median; box limits, upper and lower quartiles; whiskers are highest and lowest values no greater than 1.5× IQR.

Figure S3

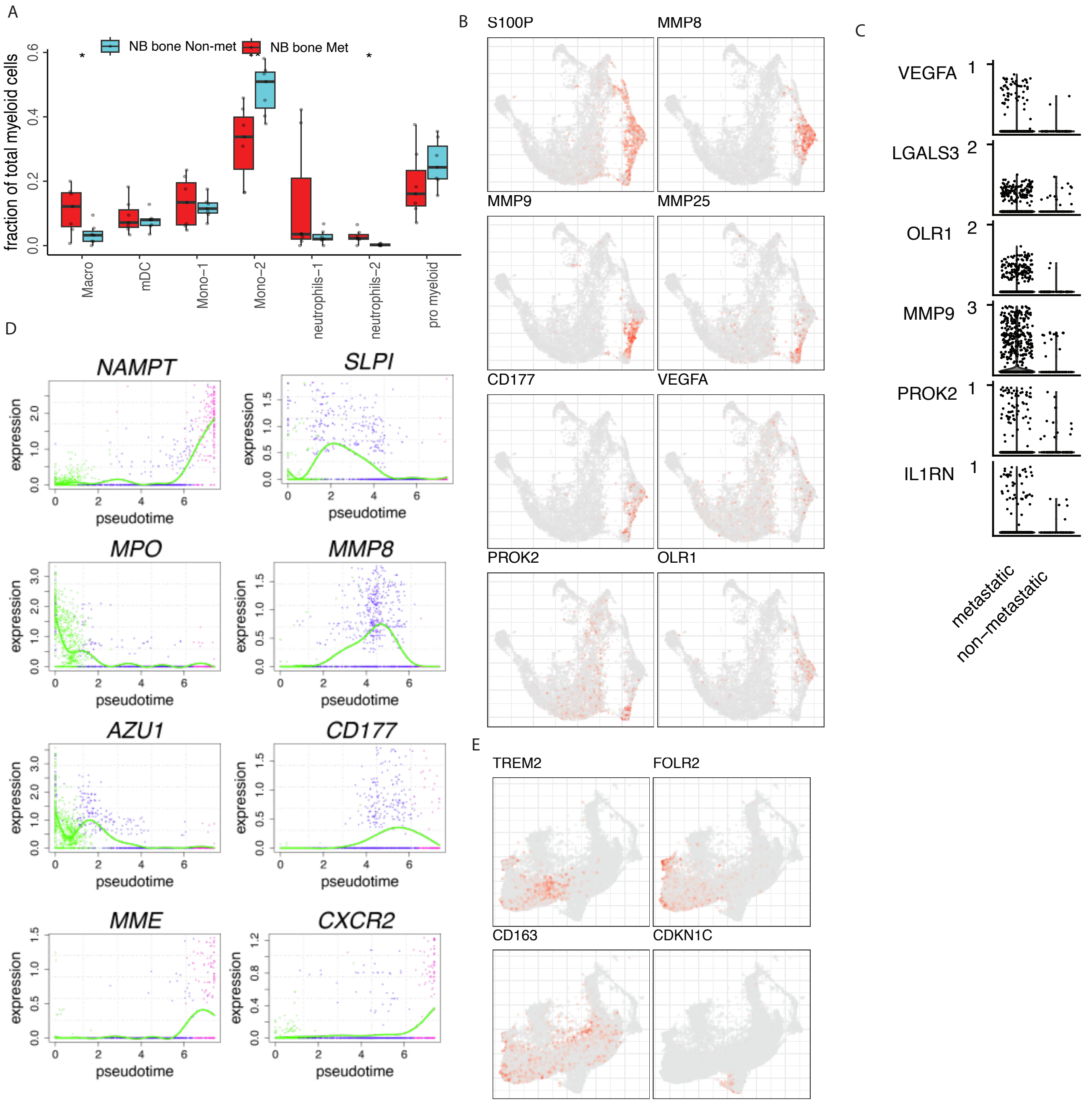

A
NB bone Non-met
NB bone Met
fraction of total myeloid cells
Macro
mDC
Mono−1
Mono−2
neutrophils−1
neutrophils−2
pro myeloid
B
S100P
MMP8
MMP9
MMP25
CD177
VEGFA
PROK2
OLR1
C
VEGFA
LGALS3
OLR1
MMP9
PROK2
IL1RN
metastatic
non−metastatic
D
NAMPT
SLPI
MPO
MMP8
AZU1
CD177
MME
CXCR2
expression
pseudotime
E
TREM2
FOLR2
CD163
CDKN1C

Supplementary Figure 3. Myeloid cells characterization, enrichment and differentiation

A. Comparison of relative cell abundance of myeloid cell subpopulations between NB bone Met (n=7) and NB bone Non-met (n=6) samples. Statistics are accessed with Wilcoxon rank sum test (*$p<0.05$, ***$p<0.001$). Boxplots include center line, median; box limits, upper and lower quartiles; whiskers are highest and lowest values no greater than 1.5× IQR.

B. Expression of selected neutrophil marker genes, visualized in myeloid UMAP embedding.

C. Violin plot showing representative marker gene expression in neutrophils.

D. Similar to Figure 2F, showing the representative gene expression along with pseudo-time moving from progenitor myeloid cells to neutrophils-1 and neutrophils-2.

E. UMAP embedding showing selected gene expression in tissue resident macrophage and bone marrow derived macrophage.

Figure S4

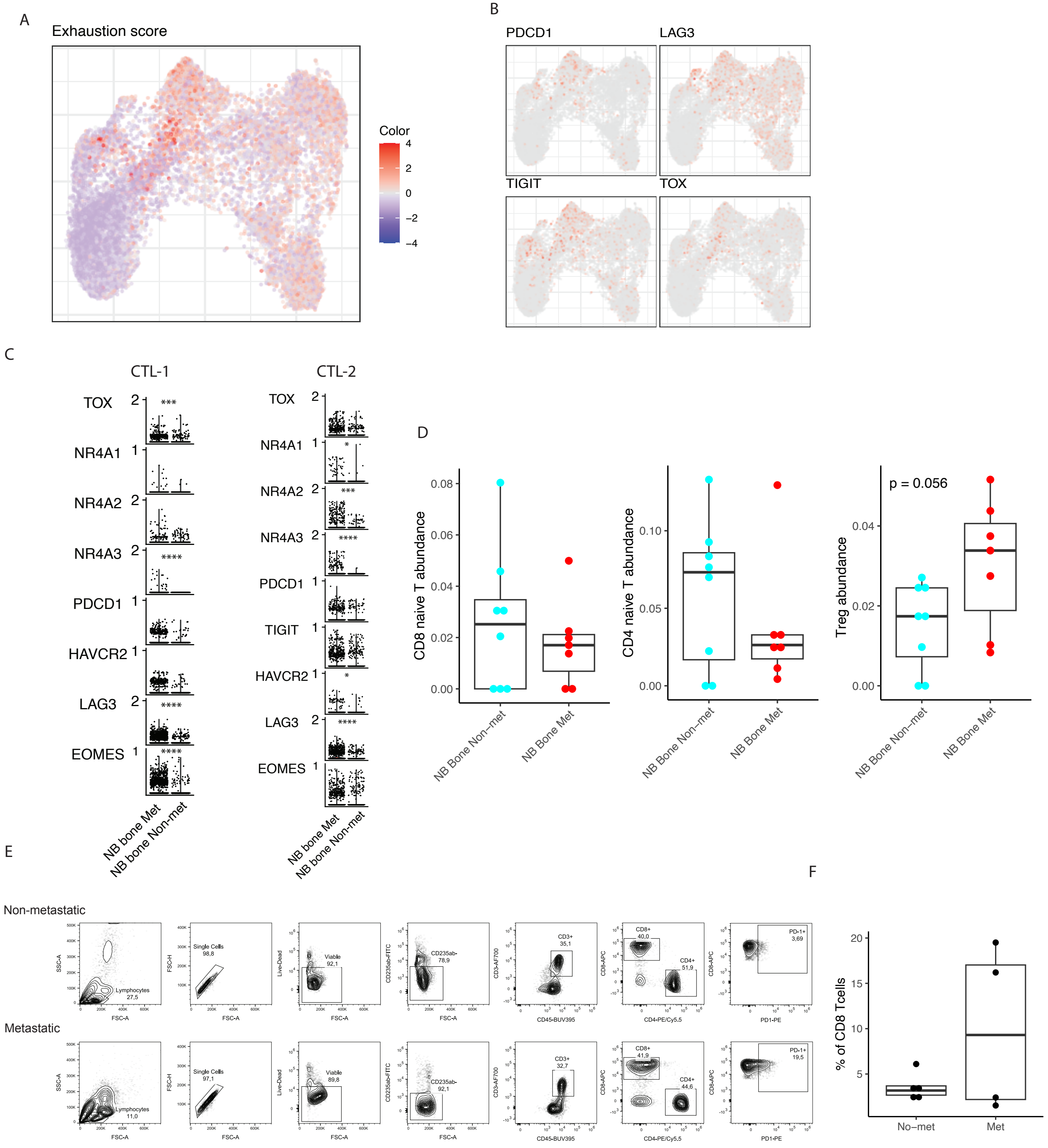
A
Exhaustion score
Color
4
2
0
−2
−4
B
PDCD1
LAG3
TIGIT
TOX
C
CTL-1
CTL-2
TOX
NR4A1
NR4A2
NR4A3
PDCD1
HAVCR2
LAG3
EOMES
TIGIT
***
****
*
NB bone Met
NB bone Non-met
D
CD8 naive T abundance
CD4 naive T abundance
Treg abundance
p = 0.056
NB Bone Non−met
NB Bone Met
E
Non-metastatic
Metastatic
Lymphocytes
27,5
11,0
Single Cells
98,8
97,1
Viable
92,1
89,8
CD235ab-
78,9
92,1
CD3+
35,1
32,7
CD8+
40,0
41,9
CD4+
51,9
44,6
PD-1+
3,69
19,5
SSC-A
FSC-A
FSC-H
Live-Dead
CD235ab-FITC
CD3-AF700
CD45-BUV395
CD8-APC
CD4-PE/Cy5,5
PD1-PE
F
% of CD8 Tcells
No−met
Met

Supplementary Figure 4. Increased abundance of tumor-infiltrating CTLs and Tregs in NB bone metastasis tumor

A. UMAP visualization of average gene expression of T cell exhaustion signatures.

B. UMAP visualization showing *PDCD1*, *LAG3*, *TIGIT* and *TOX* expression.

C. Violin plot showing representative T cell exhaustion signature genes expression in CTL-1 and CTL-2, stratifying cells by non-metastatic and metastatic samples.

D. Comparison of CD4 naïve T, CD8 naïve T and Tregs proportion in NB bone Met (n=7) and NB bone Non-met (n=7) samples. Statistics are accessed with Wilcoxon rank sum test(*$p<0.05$). Data are expressed as mean ± SEM.

E. Gating strategy for PDCD1+ CD8+ T cells. Labels above the flow plots refer to the parent population in the percentages are of the parent gate.

F. Boxplot showing the percent of PDCD1+ CD8+ T cells in NB bone Met and NB bone Non-met samples (n=3).

Figure S5

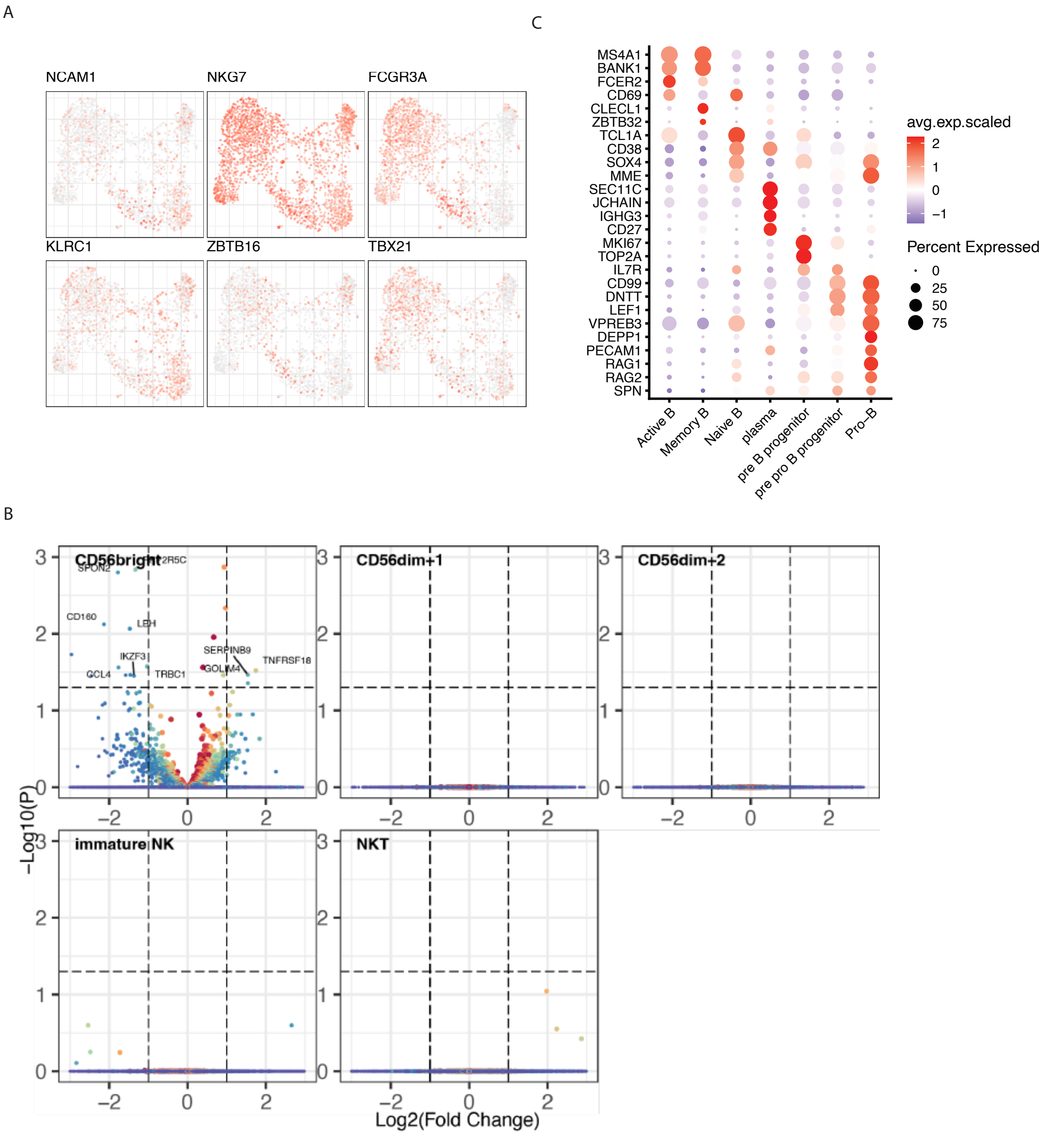

A
NCAM1
NKG7
FCGR3A
KLRC1
ZBTB16
TBX21
C
MS4A1
BANK1
FCER2
CD69
CLECL1
ZBTB32
TCL1A
CD38
SOX4
MME
SEC11C
JCHAIN
IGHG3
CD27
MKI67
TOP2A
IL7R
CD99
DNTT
LEF1
VPREB3
DEPP1
PECAM1
RAG1
RAG2
SPN
Active B
Memory B
Naive B
plasma
pre B progenitor
pre pro B progenitor
Pro–B
avg.exp.scaled
2
1
0
−1
Percent Expressed
0
25
50
75
B
CD56bright
CD56dim+1
CD56dim+2
immature NK
NKT
SPON2
CD160
LEH
IKZF3
CCL4
TRBC1
SERPINB9
GOLM4
TNFRSF18
−Log10(P)
Log2(Fold Change)

Supplementary Figure 5. Characterization of B and NK subpopulations

A. Representative NK cell marker gene expression on joint UMAP embedding.

B. Volcano plot of differential expressed genes for each NK cell subpopulations comparing metastatic samples with non-metastatic samples.

C. Dot plot demonstrating marker gene expression across different B cell subpopulations. The color represents scaled average expression of marker genes in each cell type, and the size indicates the proportion of cells expressing marker genes.

Figure S6

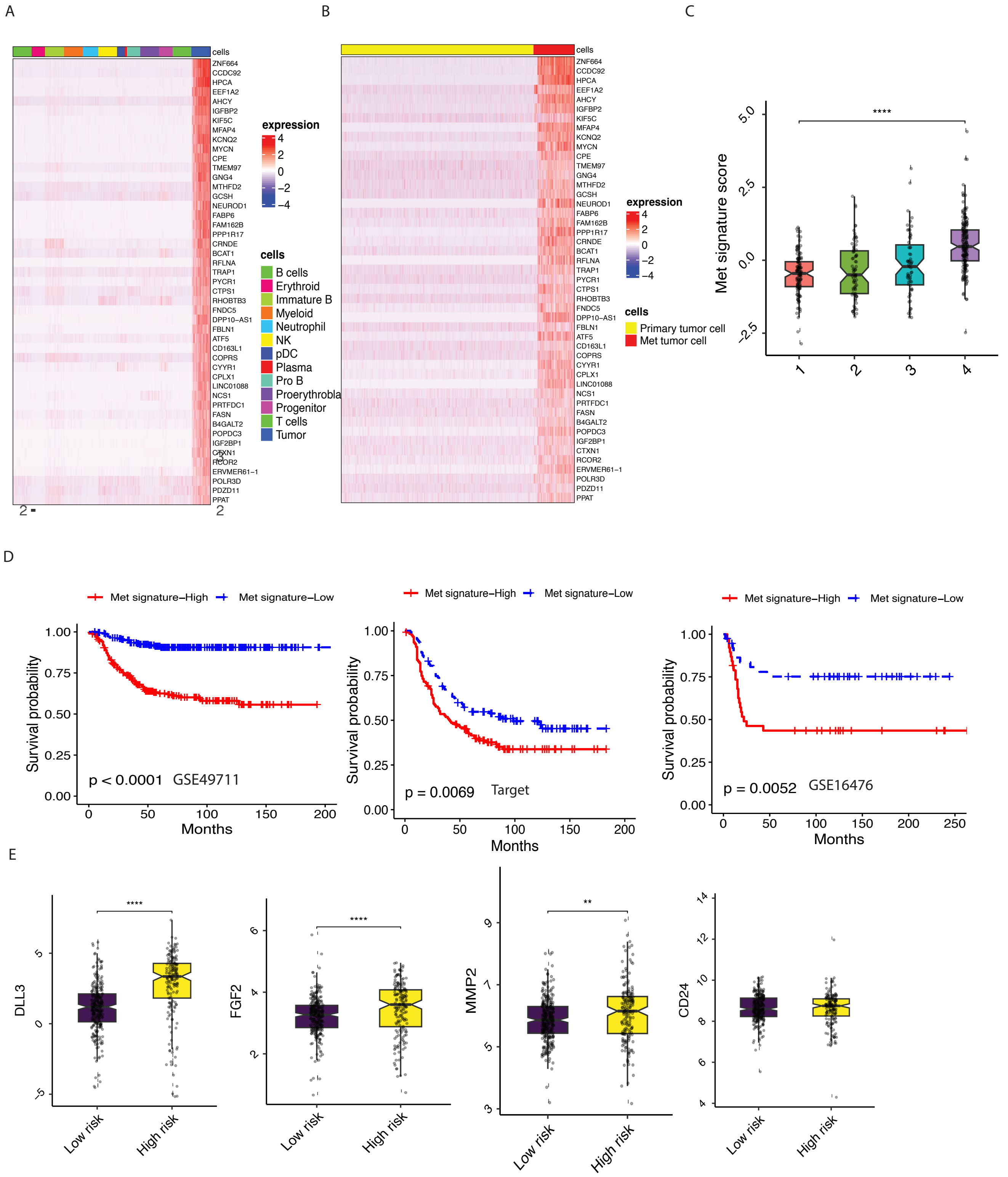

A
B
C
cells
ZNF664
CCDC92
HPCA
EEF1A2
AHCY
IGFBP2
KIF5C
MFAP4
KCNQ2
MYCN
CPE
TMEM97
GNG4
MTHFD2
GCSH
NEUROD1
FABP6
FAM162B
PPP1R17
CRNDE
BCAT1
RFLNA
TRAP1
PYCR1
CTPS1
RHOBTB3
FNDC5
DPP10−AS1
FBLN1
ATF5
CD163L1
COPRS
CYYR1
CPLX1
LINC01088
NCS1
PRTFDC1
FASN
B4GALT2
POPDC3
IGF2BP1
CTXN1
RCOR2
ERVMER61−1
POLR3D
PDZD11
PPAT
expression
4
2
0
−2
−4
cells
B cells
Erythroid
Immature B
Myeloid
Neutrophil
NK
pDC
Plasma
Pro B
Proerythrobla
Progenitor
T cells
Tumor
Primary tumor cell
Met tumor cell
Met signature score
****
1
2
3
4
D
Met signature−High
Met signature−Low
Survival probability
Months
p < 0.0001 GSE49711
p = 0.0069 Target
p = 0.0052 GSE16476
E
DLL3
FGF2
MMP2
CD24
****
**
Low risk
High risk

Supplementary Figure 6: Metastatic signature predicts neuroblastoma patient survival

A. Heatmap showing metastatic signature gene expression in major cell types in NB bone metastatic scRNAseq data.

B. Similar to Figure 5C, showing metastatic signature gene expression in NB bone metastatic and NB primary tumor cells.

C. Boxplot representing metastatic signature score in bulk RNA-seq of NB patients (GSE49711), stratifying patients by INSS stage (stage1=121, stage2=78, stage1=63, stage1=183). Significance was assessed using Wilcoxon rank sum test and BH multiple testing correction (***p<0.001).

D. Kaplan –Meier survival curves showing NB patients with higher metastatic signature gene expression have worse overall survival in three independent NB datasets (GSE49711 n=488, Target n=247, GSE16476 n=76). Patients were stratified into two groups based on the average expression (binary: top 25% versus bottom 25%) of metastatic signatures as annotated by key marker genes in panel. Statistics are accessed by two-side log-rank test.

E. Boxplot representing *DLL3*, *FGF2*, *MMP2* and *CD24* expression in low-risk (n=273) and high-risk (n=172) NB patients (GSE49711). Significance was assessed using two-sided Wilcoxon rank sum test (***p<0.01, ***p<0.001). Boxplots include center line, median; box limits, upper and lower quartiles; whiskers are highest and lowest values no greater than 1.5× IQR.
